# Rain rate modeling of 1-min from various integration times in South Korea

**DOI:** 10.1186/s40064-016-2062-3

**Published:** 2016-04-12

**Authors:** Sujan Shrestha, Jung-Jin Park, Dong-You Choi

**Affiliations:** Department of Information and Communications Engineering, Chosun University, Kwangju, Republic of Korea; Department of Photoelectronics, Chosun University, Kwangju, Republic of Korea

**Keywords:** 1-min Rain rate, Microwave radio propagation meteorological statistics, ITU-R 837-6 model

## Abstract

Rain plays a major impairment factor for propagation of electromagnetic waves in atmosphere for systems operating at frequencies above 10 GHz. Several effects are noted such as depolarization, scintillation, interference due to scattering and extra attenuation which seems to increase with frequency. To mitigate its effect in satellite communication, knowledge of local rainfall statistics is necessary which act as milestone for design of radio link. Rain attenuation is best visualize by the 1-min rainfall rate statistic but the measurement of this rain rate distribution is rare on a worldwide basis and observation of rain rate are done with longer integration times typically 30 min or more. In this paper, efforts have been made to develop model that can convert rain rate complementary cumulative distribution function to shorter integration times. The average relative error margin of about 5, 14, 43, 71 and 115 % are noted for 5 to 1-, 10 to 1-, 20 to 1, 30 to 1- and 60 to 1-min respectively from ITU-R P.837-6 method which have been analyzed in further section of this article. The empirical natures of conversion methods as such Segal method, Burgueno’s method, Chebil and Rahman method and Logarithmic model are studied along with the proposed new model that seems to be applicable in derivation of 1-min rain rate of the South Korea rain rate statistics. International Telecommunication Union-Radio communication Sector (ITU-R) has developed a recommendation ITU-R P.837-6 that enables the user to estimate the local 1-min rainfall rate statistical distribution which is compared with calculated 1-min rain rate distribution from experimental 1-min rainfall accumulation. Unfortunately, ITU-R P.837-6 estimated 1-min values show greater error percentages. In order to get better approximation of local 1-min rain rate estimation, a novel method is proposed and it’s efficiency have been compared with rainfall rate statistics obtained from nine different locations in the South Korea.

## Introduction

Due to increased congestion of communication spectrum below 10 GHz, there is an increasing need for the use of the short centimeter and millimeter wave parts of the spectrum in both terrestrial and satellite communication paths. Rain as the main attenuation factor in the microwave radio links has been recognized for more than decades mainly by telecommunication expertise. The reliable statistical modeling of the distribution of rain rate is still a matter of research. Statistical analyses and techniques are most useful for evaluation of transmission impairments on communication links (Ippolito 2012). The approach used in this study model the long term behavior of rain by analyzing the whole set of data without attempting to classify it according to event types or rain intensity. For the prediction of rain attenuation, accurate knowledge of 1-min integrated CCDF of rain rate is required as recommended by Series (2015). But difficulty to obtain such short interval rain rate have forced researcher worldwide to find prediction method that shall be able to predict local rain rate characteristics (Aris et al. 2013). Paper proposed a prediction model based on the rainfall data collected by Korea Meteorological Administration (KMA) which has developed and operated a digital system for collecting and storing rainfall data at 1-min interval since 2004. Performance of proposed model is compared with ITU-R P.837-6 (2012) and found to give better prediction performance as it makes reduction in error analysis. The material of the paper is covered as follows: “Background” section briefly introduces background of established relationships for derivation of 1-min rain rate. “Methodology and analyses of experimental data” section deals with experimental data collection procedure. “Results and discussion” section presents the result and discussion of statistical analyses. Finally, “Conclusion” section draws conclusion of highly reliable statistical results.

## Background

Rainfall data of longer integration time relatively on hourly basis is readily available, 5- or 10-min accumulation data are recorded by several weather services, but 1-min accumulation interval data is available from only special observations at a few locations. Under this scenario, derivation of model for statistical distribution of short time interval requires the limited number of available observation to be combined in order to provide statistically valid empirical distribution function (Crane 1996). As the earlier approach is presented by ITU-R with latest global model (2012), its usefulness is depicted by Emiliani et al. (2010), Capsoni and Lorenzo (2009), Capsoni et al. (2009) which highlight the benefits of using EXCELL RSC physical approach to the conversion of rainfall statistics. Furthermore, several methods based on physical approach and empirical ways are studied but when physical approach is used, all the input parameters needed for analysis is unavailable. Similarly, mathematical theory with is based on first principles for de-integrating T-min experimental probability distribution (PD) into corresponding 1-min PD are studied (Matricciani 2011). However, the contribution present the need for more efficient propagation planning, based on the use of number and duration of rain events along with the fraction of rainy time. The experimental system carried out by KMA provides the record for only experimental 1-min rainfall amount as discussed in further section of this paper. Due to the ease of simplicity and easier analysis purpose, empirical nature of rainfall rate method is chosen (Emiliani et al. 2009). The rain rate characteristic of the South Korea was studied to predict 1-min rain rate statistic in Jung et al. (2008), which was based on the 2 years of rain events. Unfortunately, it was found to be less effective and generated higher error percentage. Similarly, with three years of rainfall data, a conversion method for rainfall rate with various integration time was proposed (Lee et al. 1994) base on linear and logarithmic approach. In addition, the global empirical approach was also analyzed in Jung et al. (2008), which was compared with older version of ITU-R P.837-5 rain rate model. A model for rain drop size distribution (Park et al. 2002), was introduced which describe extended gamma distribution function. In this scenario, this paper presents a novel work for prediction of 1-min rainfall rate. Empirical methods is equally been studied in other countries as noted in Ong and Zhu (1997), Singh et al. (2006, 2007), Mandeep and Hassan (2008), Segal (1986), Burgueno et al. (1988) from long term rainfall database. In addition, the global coefficients values are listed in Emiliani et al. (2008) which extend its application to rain rate conversion methods in temperate, tropical and cold climates.

The performance of proposed ITU-R P.837-6 (2012) method for derivation of 1-min rain rate is compared with globally applicable empirical methods namely Segal (1986), Burgueno et al. (1988), Chebil and Rahman (1999), logarithmic (Lee et al. 1994) along with global coefficients approach and polynomial fit analysis of the rainfall rate data. The use of polynomial relationship was found to be better for derivation of 1-min integration time as shown in Khairolanuar et al. (2014), Owolawi (2011). The brief overviews of applied models are presented below:

### Segal method

This method was developed based on database of high resolution rainfall records prepared at the Communications Research Centre. The rainfall records were taken from ten years of daily tipping bucket rain gauge charts for each of the 47 stations in Canada. The conversion method is expressed as (Segal 1986):

1 $${\mathbf{R}}_{1} ({\mathbf{P}}) = {\varvec{\uprho}}_{{\varvec{\uptau}}} ({\mathbf{P}}){\mathbf{R}}_{{\varvec{\uptau}}} ({\mathbf{P}})$$

where, conversion factor, **ρ**_**τ**_**(P)** = **aP**^**b**^, **R**_**1**_(**P**) represents the rainfall rate in a 1-min integration time with the possibility of occurrence P, **R**_**τ**_**(P)** is the rainfall rate in τ-minutes integration time, and parameters a and b are regression coefficients that are derived from statistical analysis of rainfall data.

### Burgueno et al. method

Based on 49 years of rainfall data measured at Barcelona, Spain, Burgueno et al used direct power law fit as (Burgueno et al. 1988):

2 $${\mathbf{R}}_{1} ({\mathbf{P}}) = {\mathbf{aR}}_{{\varvec{\uptau}}}^{{\mathbf{b}}} ({\mathbf{P}})$$

where R_1_(P) and R_τ_(P) are the precipitation rates with a sampling interval of 1- and τ-min respectively with equal probability of time percentage P, a and b represent the conversion variables.

### Chebil and Rahman method

Chebil and Rahman introduced an experimental technique for estimating the precipitation rate conversion element by using the conversion process from 60- to 1-min integration time as (Chebil and Rahman 1999):

3 $${\varvec{\uprho}}_{60} ({\mathbf{P}}) = {\mathbf{R}}_{1} ({\mathbf{P}})/{\mathbf{R}}_{60} ({\mathbf{P}})$$

where **R**_**60**_(**P**) is the precipitation rate in 60-min integration time. **ρ**_**60**_(**P**) is expressed as a mixed power-exponential law, **ρ**_60_(**P**) = **aP**^**b**^ + **ce**^(**dP**)^ with regression variables represented by a, b, c and d analyzed from statistical analysis of rainfall data. Suitability of this method has been further tested for other lower integration time intervals.

### Logarithmic model

The expression for this model is given as (Lee et al. 1994):

4 $${\mathbf{log}}\left[ {{\mathbf{R}}_{1} ({\mathbf{P}})} \right] = {\mathbf{a}}\,{\mathbf{log}}\left[ {{\mathbf{R}}_{{\varvec{\uptau}}} ({\mathbf{P}})} \right]$$

where a is the regression variable derived from statistical analysis of rainfall rate.

### ITU-R recommended model

The most acknowledge model of the latter kind included in Study Group 3 Report for the update of Rec. ITU-R P.837-5, Annex 3 (2012), which allows global 1-min rain rate prediction from the knowledge of the local rainfall rate. EXCELL Rainfall Statistics Conversion (ERSC) (henceforth EXCELL RSC) method is used for conversion of rainfall rate statistics from long to 1-min integration time. This method is based on the simulated movement of rain cells over a virtual rain gauge, with given integration time T, whose translation velocity depends both on the type of precipitation and on the observation period. The conversion of rainfall was obtained using a virtual rain gauge according to the local mean yearly wind velocity, which as extracted from the ERA-40 database. The model goes through an iterative inversion procedure that aims at identifying the local P(R)_1_ (a set of P_0_, n, R_a_) which when used as input to the rain gauge simulator, provides the best possible estimate of the measured P(R)_T_. The detail of this approach can be obtained from description of method adopted for the update of Rec. ITU-R P.837-5, Annex 3.

### Proposed model

The new model is based on the curve fitting technique analyze from Matlab. Polynomial in one variable is expressed as ${\text{a}}_{\text{n}} {\text{x}}^{\text{n}} + {\text{a}}_{{{\text{n}} - 1}} {\text{x}}^{{{\text{n}} - 1}} + \cdots + {\text{a}}_{1} {\text{x}}^{1} + {\text{a}}_{0}$ where x is a variable and exponents are non negative integers with real number coefficients and ${\text{a}}_{\text{n}} \ne 0.$ A function in the form ${\text{f}}({\text{x}}) = {\text{a}}_{\text{n}} {\text{x}}^{\text{n}} + {\text{a}}_{{{\text{n}} - 1}} {\text{x}}^{{{\text{n}} - 1}} + \cdots + {\text{a}}_{1} {\text{x}}^{1} + {\text{a}}_{0}$ is a polynomial function. Rain cells characteristics have been found to be better shown by an exponential profile which is able to represent real single-peaked rain structure (Luini and Capsoni 2011). The rainfall pattern is observed to be visible through exponential coefficients. This paper present a comparison with several polynomial functions and proposed **Model1** of modified fourth order polynomial function as a suitable approach for South Korea’s own numerical prediction model for 1-min rainfall rate derivation. These models are represented as:

5 $${\mathbf{Model}}\,{\mathbf{1}}\,{\text{is}}\,{\text{expressed}}\,{\text{as}}\,{\mathbf{R}}_{{\mathbf{1}}} ({\mathbf{P}}) = {\mathbf{ae}}^{{({\mathbf{a}} - {\mathbf{b}})}} [{\mathbf{R}}_{{\varvec{\uptau}}} ({\mathbf{P}})]^{4} + {\mathbf{be}}^{{({\mathbf{b}} - {\mathbf{a}})}} [{\mathbf{R}}_{{\varvec{\uptau}}} ({\mathbf{P}})]^{3} + {\mathbf{c}}[{\mathbf{R}}_{{\varvec{\uptau}}} ({\mathbf{P}})]^{2} + {\mathbf{d}}[{\mathbf{R}}_{{\varvec{\uptau}}} ({\mathbf{P}})]$$

6 $${\mathbf{Model}}\,{\mathbf{2}}\,{\text{is}}\,{\text{expressed}}\,{\text{as}}\,{\mathbf{R}}_{{\mathbf{1}}} ({\mathbf{P}}) = {\mathbf{ae}}^{{({\mathbf{a}} - {\mathbf{b}})}} [{\mathbf{R}}_{{\varvec{\uptau}}} ({\mathbf{P}})]^{2} + {\mathbf{be}}^{{({\mathbf{b}} - {\mathbf{a}})}} [{\mathbf{R}}_{{\varvec{\uptau}}} ({\mathbf{P}})]$$

7 $${\mathbf{Model}}\,{\mathbf{3}}\,{\text{is}}\,{\text{expressed}}\,{\text{as}}\,{\mathbf{R}}_{{\mathbf{1}}} ({\mathbf{P}}) = {\mathbf{ae}}^{{({\mathbf{a}} - {\mathbf{b}})}} [{\mathbf{R}}_{{\varvec{\uptau}}} ({\mathbf{P}})]^{2} + {\mathbf{be}}^{{({\mathbf{b}} - {\mathbf{a}})}} [{\mathbf{R}}_{{\varvec{\uptau}}} ({\mathbf{P}})] + {\mathbf{c}}$$

8 $${\mathbf{R}}_{{\mathbf{1}}} ({\mathbf{P}}) = {\mathbf{a}}\left[ {{\mathbf{R}}_{{\varvec{\uptau}}} ({\mathbf{P}})} \right]^{2} + {\mathbf{b}}\left[ {{\mathbf{R}}_{{\varvec{\uptau}}} ({\mathbf{P}})} \right] + {\mathbf{c}},\,{\text{represent}}\,{\text{second}}\,{\text{degree}}\,{\text{polynomial}}\,{\text{function}}$$

9 $${\mathbf{R}}_{{\mathbf{1}}} ({\mathbf{P}}) = {\mathbf{a}}\left[ {{\mathbf{R}}_{{\varvec{\uptau}}} ({\mathbf{P}})} \right]^{3} + {\mathbf{b}}\left[ {{\mathbf{R}}_{{\varvec{\uptau}}} ({\mathbf{P}})} \right]^{2} + {\mathbf{c}}\left[ {{\mathbf{R}}_{{\varvec{\uptau}}} ({\mathbf{P}})} \right] + {\mathbf{d}},\,{\text{represent}}\,{\text{third}}\,{\text{degree}}\,{\text{polynomial}}\,{\text{function}}$$

where **R**_1_(**P**) represents the rainfall rate in a 1-min integration time with the possibility of occurrence P, **R**_**τ**_**(P**) is the rainfall rate in τ-minutes integration time, and coefficients a, b, c and d are regression coefficients that are obtained through statistical analysis of rainfall data with the use of curve fitting technique derived from Matlab programming. The effectiveness of proposed Model 1 are measured through various error analyses which highlight better agreement with experimental 1-min rainfall data obtained from KMA for listed nine different regions of South Korea. The importance of constant values and modification on coefficients are judged through regression analysis and curve fitting approach which give minimum error values against the calculated 1-min rain rate values from experimental rainfall amount collection under 1-min duration.

## Methodology and analyses of experimental data

The Republic of Korea lies in temperate zone with four distinct seasons. Geographically, country is located in the middle latitudes of the Northern Hemisphere, on the east coast of the Eurasian Continent and also adjacent to the Western Pacific. Thus, complex climate characteristics are observed in this belt which reveals both continental and oceanic features. The seasonal climate characteristic of the South Korea is shown in Table 1.

**Table 1:**
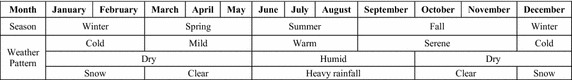
Seasonal climatic characteristic

**Fig. 1:**
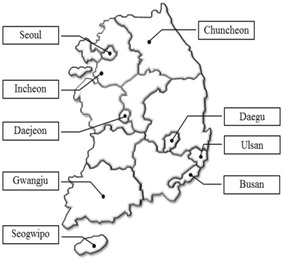
Major cities of the South Korea

Rainfall data is effectively recorded by KMA, a central governmental organization of the Republic of Korea under the Ministry of Environment (MOE), which has developed a digital system for accurate measurement of 1-min rainfall amount since 2004 through the use of Tipping bucket rain gauge over several sites some of which are shown in Fig. 1 with the intensity to develop the South Korea’s own numerical prediction model.

Furthermore, Table 2 indicates the selected sites with their geographical co-ordinate values.

**Table 2:** Locations selected for model testing

Climatic region	Station location	Latitude (N)	Longitude (E)	Measurement duration (years)
Temperate	Gwangju	35.16	126.86	10
	Daegu	35.87	128.6	
	Daejeon	36.35	127.39	
	Busan	35.18	129.08	
	Seogwipo	33.25	126.56	
	Seoul	37.56	126.99	
	Ulsan	35.54	129.31	
	Incheon	37.45	126.73	
	Chuncheon	37.88	127.73	

Tipping bucket rain gauge, which is used throughout the measurement sites, automatically record rainfall and facilitate the digitization of telemetric observation signals. The heater is installed inside the sewer for measurement under snowfall.

**Fig. 2:**
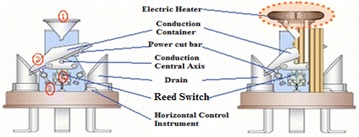
Tipping bucket rain gauge

Figure 2 shows the internal structure of rain gauge. KMA uses conducting vessel size of 0.5 mm to improve the shortcomings of the gutter. Once the collected water is more than 0.5 mm it eventually, fills the bucket. Bucket is mounted in particular axis of rotation to shift the center like a seesaw. This bucket is in contact with the Reed Switch with the rotation axis which is operated by electrical pulse occurred due to tipping phenomenon. Finally, signal generated through Reed Switch is recorded on recording device which provide measurement of 1-min rainfall amount.

Table 3 shows specification of rain gauge used for recording of 1-min rainfall amount.

**Table 3:** Specification of tipping bucket rain gauge

Equipment	Specification	Description
Rain gauge	Sensor type	Tipping bucket
	Switch	Form A reed, mercury-wetted
	Size	200 mm in diameter
	Resolution	0.5 mm
	Sensitivity	0.1 mm per tips
	Accuracy	<5 %
	Operating temperature	−40 to +50 °C

**Fig. 3:**
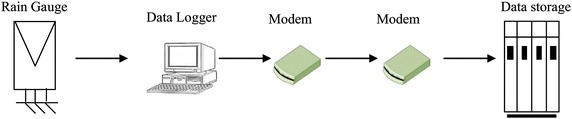
Experimental system used for measuring 1-min rainfall amount

The tipping bucket has unstably balanced twin-bucket with sensitivity of 0.1 mm per tips which trigger an electronic impulse and is stored in the data logger which scans the data at an interval over 1-min. The availability of the gauge is about 99.2 %. The 0.8 %unavailability is due to system maintenance. Figure 3 shows the overall operation of experimental system used for rainfall amount data logging parts where the accumulated rainfall amount data is first collected in a data logger which record the number of tips for every 1-min interval which is then converted to rainfall amount and finally stored in data storage devices.

ITU rain attenuation prediction method, Series (2015), is based on 0.01 % of a time for rain rate parameter. Similarly, Recommendations ITUR (2015) emphasizes on use of rain rate exceeded for 0.01 % of the time with an integration time of 1-min for long term statistics of rain attenuation. The importance of 1-min rain rate has been further studied for satellite and terrestrial rain attenuation predictions (Abdulrahman et al. 2010; Islam et al. 2012). The calculated values for rainfall rate at 0.01 % of the annual time percentage were 90, 60, 79.8, 90, 90, 90, 65.4, 89.4 and 60 mm/h over 1-min integration time for Gwangju, Daegu, Daejeon, Busan, Seogwipo, Seoul, Ulsan, Incheon and Chuncheon sites respectively. Unfortunately, ITU-R 837-6 (2012) model prediction overestimate the rain rate of these mentioned sites and does not satisfies the local statistical data. The received decade 1-min rainfall amounts are arranged for other time integrations period as 5-, 10-, 20-, 30- and 60-min. These data are sorted and required rainfall amount are extracted for several times percentages as mentioned by ITU-R P.311-15 (2015). For example, for Gwangju site, at 0.01 % of time, 1-min rainfall amount for 526 ((10 × 365 × 24 × 60 × 0.01)/100) instance was taken which is converted to rain rate expression as mentioned in Kestwal et al. (2014). The result of 1-, 5-, 10-, 20-, 30- and 60-min rainfall rate of selected nine major cities as Gwangju, Daegu, Daejeon, Busan, Seogwipo, Seoul, Ulsan, Incheon and Chuncheon, are calculated from experimental 1-min rainfall amount as obtained from KMA which are summarized in Table 4. These data are used as a basis for comparison with estimated 1-min rain rate from ITU-R P.837-6 (2012) and to propose local prediction model. In order to generate estimated 1-min rain rate from the software as recommended by ITU-R P.837-6 Annex 3, we have selected Mode A operational mode in which rain rate values for several source integration times as stated in Table 4 along with sites latitude and longitude information as mentioned in Table 2 is given as an input source data. The T-min integrated percentage values are 0.1, 0.05, 0.03, 0.02, 0.01, 0.005, 0.003, 0.002, and 0.001 %. Since the calculated rain rate values for greater time percentages beyond 0.1 % is very low and tend to be negligible, so we have not included for analyses purpose.

**Table 4:** Calculated rainfall rate statistics over various integration times (unit: mm/h)

Station ID	Time percentage	1-min	5-min	10-min	20-min	30-min	60-min
Gwangju	0.1	45.60	42.96	42.06	41.52	40.56	38.11
	0.05	60.00	50.88	49.26	47.88	47.04	43.82
	0.03	60.00	57.72	55.68	52.77	51.36	48.79
	0.02	63.60	63.48	60.00	56.34	56.28	51.34
	*0.01*	*90.00*	*72.00*	*69.78*	*65.58*	*65.30*	*60.46*
	0.005	90.00	84.00	78.00	74.13	74.82	72.30
	0.003	105.60	90.00	83.94	79.59	79.52	72.56
	0.002	120.00	102.00	90.00	82.50	84.00	72.57
	0.001	120.00	114.00	102.00	93.00	94.00	74.10
Daegu	0.1	38.40	36.48	36.00	35.34	35.12	34.32
	0.05	43.20	41.16	39.60	38.91	38.16	37.28
	0.03	49.20	43.80	42.42	41.34	40.46	39.00
	0.02	60.00	48.00	45.12	43.68	42.78	41.16
	*0.01*	*60.00*	*54.00*	*51.00*	*47.70*	*45.00*	*43.54*
	0.005	90.00	72.00	60.00	55.50	49.00	46.73
	0.003	90.00	78.00	69.00	60.00	54.00	47.50
	0.002	90.00	84.00	75.00	67.50	56.00	47.73
	0.001	120.00	102.00	96.00	81.00	84.00	62.50
Daejeon	0.1	41.40	38.88	38.16	37.26	36.48	35.72
	0.05	53.40	45.48	43.80	42.00	40.78	39.23
	0.03	60.00	54.00	51.00	48.00	45.68	43.14
	0.02	60.00	60.12	58.44	56.97	56.48	52.50
	*0.01*	*79.80*	*69.36*	*66.36*	*63.27*	*63.26*	*61.74*
	0.005	90.00	78.00	75.00	68.28	65.38	64.22
	0.003	90.00	84.00	81.00	72.00	66.98	64.39
	0.002	120.00	96.00	84.00	78.00	68.20	65.75
	0.001	120.00	114.00	117.00	88.50	73.00	67.59
Busan	0.1	57.60	55.56	55.14	54.75	54.68	53.84
	0.05	64.20	62.88	62.04	61.11	60.56	60.20
	0.03	70.20	67.68	67.14	66.24	65.44	64.57
	0.02	76.20	71.64	69.78	69.00	68.06	67.37
	*0.01*	*90.00*	*78.00*	*76.74*	*76.50*	*75.60*	*73.50*
	0.005	90.60	88.44	84.54	83.25	80.96	84.81
	0.003	108.00	96.00	90.00	87.21	86.16	85.00
	0.002	120.00	104.28	96.00	94.02	92.36	91.00
	0.001	150.00	126.00	117.00	111.00	106.00	98.50
Seogwipo	0.1	46.20	45.96	44.46	42.54	42.00	41.59
	0.05	60.00	57.84	55.26	54.00	52.10	52.70
	0.03	68.40	66.00	64.44	61.77	62.34	62.17
	0.02	78.60	72.00	69.00	67.11	66.38	66.29
	*0.01*	*90.00*	*84.00*	*77.58*	*76.32*	*77.08*	*76.33*
	0.005	100.80	96.00	94.50	97.50	91.60	95.72
	0.003	114.00	102.00	102.60	102.66	104.80	101.70
	0.002	120.00	107.52	107.58	110.52	107.26	103.23
	0.001	120.00	114.00	120.00	123.00	113.00	199.80
Seoul	0.1	48.60	46.80	45.18	44.43	43.50	42.12
	0.05	58.20	53.88	51.54	50.52	50.40	48.20
	0.03	60.00	58.32	55.80	54.60	53.34	51.08
	0.02	66.00	60.96	59.34	57.00	56.04	53.94
	*0.01*	*90.00*	*72.48*	*69.00*	*61.95*	*60.48*	*56.67*
	0.005	90.00	84.00	78.00	70.50	70.00	63.37
	0.003	120.00	90.00	87.00	76.50	72.00	65.00
	0.002	120.00	96.00	93.00	81.00	74.18	70.66
	0.001	150.00	114.00	105.00	85.50	77.00	74.00
Ulsan	0.1	44.40	42.72	42.12	41.49	41.14	40.77
	0.05	50.40	48.00	47.58	46.50	46.18	46.46
	0.03	55.80	51.84	51.00	49.53	49.22	49.18
	0.02	60.00	54.84	54.24	53.10	51.84	54.00
	*0.01*	*65.40*	*62.04*	*61.32*	*59.73*	*59.80*	*69.13*
	0.005	78.00	72.00	70.62	70.53	85.00	146.66
	0.003	90.00	84.00	81.42	376.59	264.98	152.48
	0.002	90.00	101.04	122.16	389.28	265.70	177.42
	0.001	120.00	231.60	745.74	412.74	354.84	203.42
Incheon	0.1	60.00	57.72	57.00	55.89	55.60	55.26
	0.05	66.60	64.68	63.36	62.91	63.02	62.87
	0.03	73.20	70.92	69.36	68.91	67.86	66.32
	0.02	78.60	74.52	73.02	73.47	71.50	71.39
	*0.01*	*89.40*	*80.16*	*78.30*	*77.88*	*77.20*	*76.60*
	0.005	90.00	85.44	82.62	79.23	79.92	82.27
	0.003	96.00	91.20	85.20	83.58	82.50	83.50
	0.002	106.20	96.00	90.00	86.28	82.70	83.60
	0.001	120.00	108.00	93.00	87.51	85.60	84.18
Chuncheon	0.1	31.20	30.72	30.30	30.00	29.24	28.38
	0.05	36.00	36.00	35.76	33.66	33.08	31.23
	0.03	41.40	42.00	40.74	37.50	36.00	34.97
	0.02	58.80	48.00	45.00	40.44	38.62	35.50
	*0.01*	*60.00*	*60.00*	*57.00*	*46.50*	*48.00*	*38.52*
	0.005	90.00	78.00	69.00	60.00	53.00	49.00
	0.003	90.00	90.00	75.00	69.00	63.00	51.00
	0.002	120.00	102.00	84.00	75.00	63.00	52.50
	0.001	120.00	108.00	105.00	81.00	73.00	75.20

## Results and discussion

**Fig. 4:**
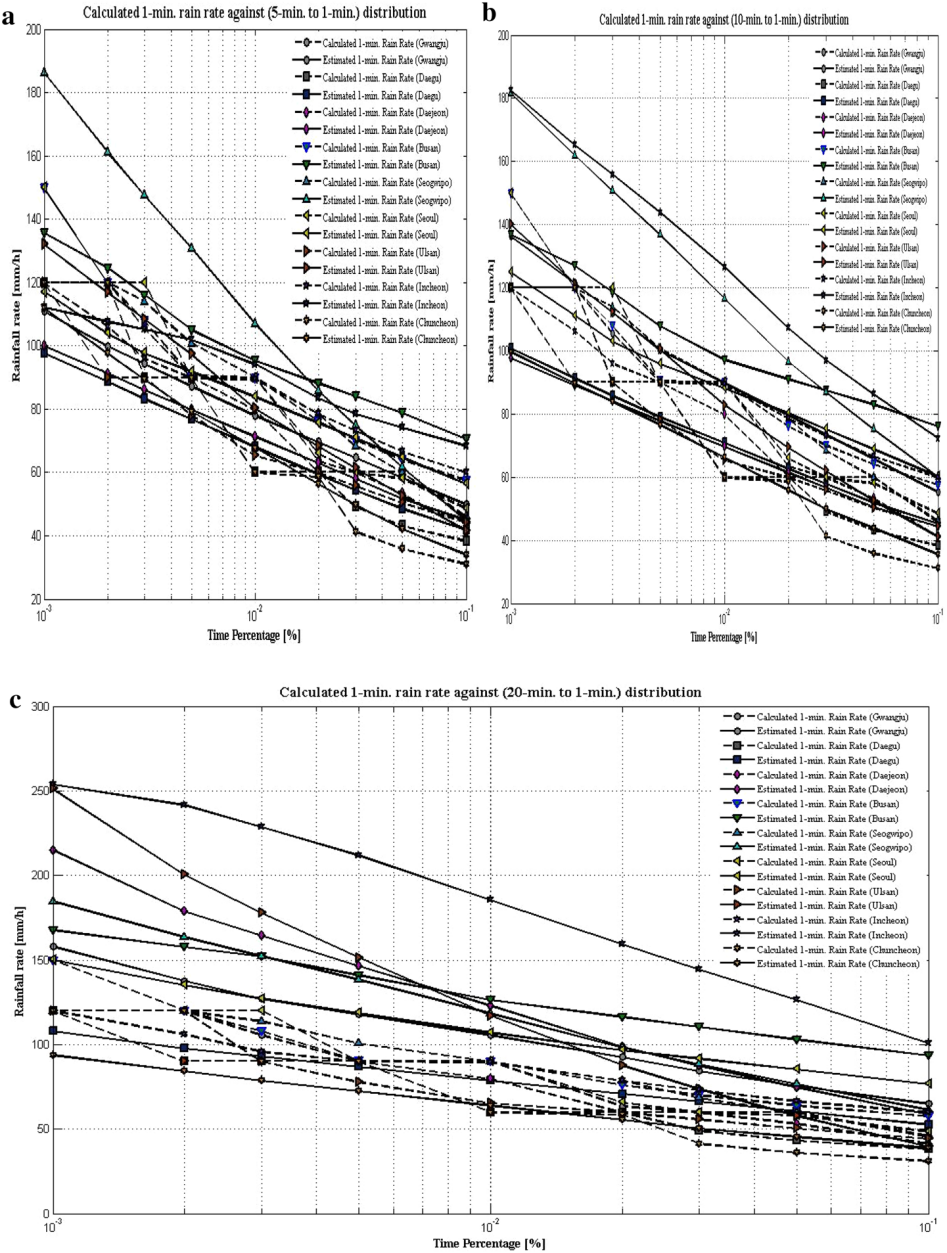
**a** Cumulative distributions of 1-min rainfall rate compared with 5-min integration rainfall rate data. **b** Cumulative distributions of 1-min rainfall rate compared with 10-min integration rainfall rate data. **c** Cumulative distributions of 1-min rainfall rate compared with 20-min integration rainfall rate data

**Fig. 5:**
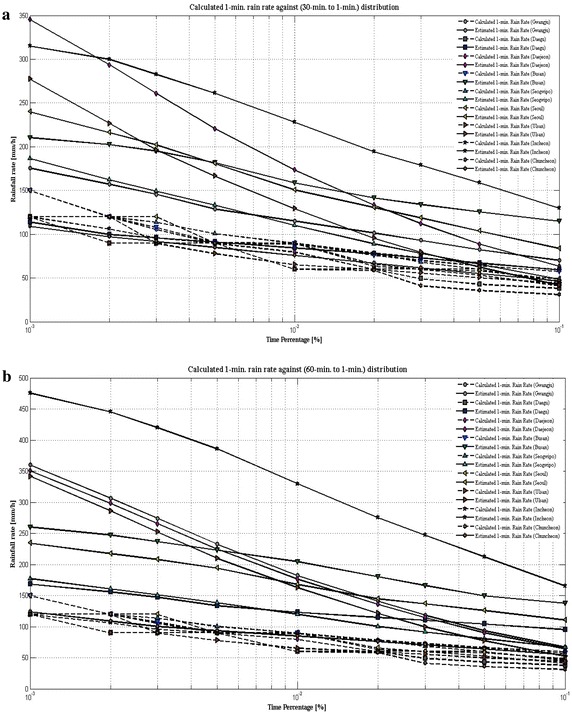
**a** Cumulative distributions of 1-min rainfall rate compared with 30-min integration rainfall rate data. **b** Cumulative distributions of 1-min rainfall rate compared with 60-min integration rainfall rate data

In order to better visualize the 1-min rain rate distribution data against ITU-R P.837-6 (2012) predicted values for several time percentage over the nine sites in the South Korea at equiprobable exceedance probability (0.001 ≤ P ≤ 0.1 %), CCDFs of rain rate are plotted which are shown from Figs. 4a–c, 5a, b. The plots include the value of Daejeon (also called as Taejon) site, which was chosen by ITU-R P.837-6 (2012) model in its ERA-40 database (Uppala et al. 2005). For the experimental purpose, Taejon site is included within Daejeon site by KMA. ITU-R P.837-6 (2012) model does not accurately predict the 1-min rain rate distribution for nine sites even though this model shows fair well statistics at lower time conversion. In addition, this model dramatically overestimate 1-min rain rate at higher time conversion. For instance, as depicted in Fig. 4a for 5- to 1-min integration time, ITU-R P.837-6 (2012) models seems to give fairy satisfactory result but chances of error is still remain, particularly for Seogwipo site. As integration times increase to 10-, 20-, 30-, and 60-min the probability of overestimating 1-min rain rate is increased as highlighted from Figs. 4b, c, 5a, b. The reason behind this difference could be the fact that the matrices used to obtain the parameters might have low spatial resolution. This indicates that ITU-R P.837-6 (2012) model performance statistics does not shows good pattern with calculated rainfall rate from experimental 1-min rainfall amount of the South Korea regions. In this concern, there is the immediate need for 1-min rain rate prediction model that can show greater efficiency against the local 1-min rain rate distribution. Under this scenario, the paper presents new model that shall be applicable in analyzing the 1-min rain fall rate distribution pattern.

As an initial step, regression analysis is performed to match the data to known distribution. Regression analysis is a statistical method to estimate the values of dependent variables that correspond to certain values of new independent variables once the magnitude of the influence of independent variables on dependent variables is measured, thereby determining the regression plane or line with regard to the independent variables. This model summarizes the large amount of data with minimum modeling error (Crane 1996). The regression coefficients applicable for mentioned nine sites are generated through curve fitting approach using Matlab programming whose generated values are listed in Tables 5, 6, 7 and 8 along with the average coefficients generated out of nine sites. These regression coefficients are considered useful for obtaining 1-min rain rate when rain rates are available at different integration times, especially when long-term precipitation data from meteorological stations are utilized for obtaining short integration time rain rates for attenuation prediction. Thus using ten rain conversion methods and coefficients from Tables 5, 6, 7 and 8, rain rates at different integration times are converted to 1-min rain rate distribution.

**Table 5:** Regression coefficients for three empirical conversion methods at different integration time

Sites	5- to 1-min		10- to 1-min		20- to 1-min		30- to 1-min		60- to 1-min	
	a	b	a	b	a	b	a	b	a	b
Segal										
Gwangju	1.0320	−0.007980	0.9034	−0.029170	0.8480	−0.04207	0.9165	−0.03419	0.7688	−0.06365
Daegu	0.9786	−0.016210	0.8909	−0.034940	0.7120	−0.06708	0.6601	−0.08183	0.4524	−0.13200
Daejeon	1.0390	−0.007406	1.0500	−0.011200	0.8047	−0.04901	0.6131	−0.08508	0.6097	−0.09043
Busan	0.8240	−0.030120	0.7169	−0.049160	0.6784	−0.05737	0.6433	−0.06488	0.6122	−0.07195
Seogwipo	0.9389	−0.013700	1.1040	0.001613	1.2870	0.01742	1.2210	0.00950	1.7560	0.05380
Seoul	0.6868	−0.056470	0.6855	−0.061760	0.4986	−0.10560	0.4149	−0.12980	0.4235	−0.13370
Ulsan	1.8630	0.069570	2.7560	0.117600	6.0760	0.22180	5.6900	0.21280	4.3240	0.18100
Incheon	0.9273	−0.015040	0.7738	−0.039800	0.7088	−0.05163	0.6968	−0.05501	0.7047	−0.05390
Chuncheon	0.8845	−0.021160	0.7442	−0.048390	0.6189	−0.08094	0.5131	−0.10880	0.5310	−0.11620
Average	1.0193	−0.010946	1.0694	−0.017245	1.3592	−0.02383	1.2632	−0.03748	1.1314	−0.04745
Burgueno et al.										
Gwangju	1.1100	1.0010	0.6884	1.1290	0.5063	1.2180	0.6372	1.1640	0.3297	1.3530
Daegu	0.9804	1.0370	1.0030	1.0540	0.4811	1.2600	0.9312	1.1120	0.0832	1.7760
Daejeon	1.1160	1.0000	1.7360	0.9068	0.4157	1.2720	0.1395	1.5610	0.1581	1.5470
Busan	0.4663	1.1930	0.2742	1.3250	0.1840	1.4220	0.1261	1.5150	0.0852	1.6110
Seogwipo	0.8069	1.0640	1.4190	0.9400	1.9810	0.8650	1.5310	0.9282	9.6970	0.4999
Seoul	0.3298	1.2930	0.2657	1.3580	0.0554	1.7640	0.0166	2.0720	0.0145	2.1370
Ulsan	8.4210	0.4972	22.5800	0.2593	19.7600	0.2734	15.0400	0.3372	7.8480	0.4883
Incheon	0.6172	1.1250	0.1828	1.4170	0.1002	1.5640	0.0798	1.6220	0.1467	1.4810
Chuncheon	0.7969	1.0730	0.8316	1.0880	0.4138	1.2970	0.2645	1.4370	0.7919	1.1910
Average	1.6272	1.0315	3.2201	1.0530	2.6553	1.2150	2.0851	1.3054	2.1283	1.3427
Model 2										
Gwangju	−0.0003004	0.6125000	0.0035520	0.5861000	0.0069850	0.5676000	0.0053480	0.5923000	0.0141600	0.5292000
Daegu	0.0010200	0.6055000	0.0013070	0.6387000	0.0105600	0.5819000	0.0038140	0.6839000	0.0350000	0.2685000
Daejeon	−0.0003225	0.6133000	−0.0038170	0.6709000	0.0094290	0.5425000	0.0194400	0.3976000	0.0212600	0.4175000
Busan	0.0039890	0.5281000	0.0070420	0.4862000	0.0092930	0.4431000	0.0113200	0.3971000	0.0132000	0.3410000
Seogwipo	0.0013580	0.5687000	−0.0019300	0.6280000	−0.0039410	0.6566000	−0.0021520	0.6391000	−0.0091750	0.7217000
Seoul	0.0071280	0.5075000	0.0095760	0.4899000	0.0197600	0.2576000	0.0215300	−0.0636200	0.0220300	−0.2340000
Ulsan	−0.0062040	0.6528000	−0.0022010	0.5904000	−0.0043060	0.6224000	−0.0033930	0.5493000	−0.0053660	0.5967000
Incheon	0.0027730	0.5443000	0.0093160	0.4248000	0.0121300	0.3511000	0.0132800	0.3203000	0.0113600	0.4003000
Chuncheon	0.0020380	0.5674000	0.0019920	0.6131000	0.0123500	0.5529000	0.0204000	0.5059000	0.0096580	0.7083000
Average	0.0012755	0.5777889	0.0027597	0.5697889	0.0080289	0.5084111	0.0099541	0.4468756	0.0124586	0.4165778

**Table 6:** Regression coefficients for Logarithmic model at different integration time

Sites	5- to 1-min	10- to 1-min	20- to 1-min	30- to 1-min	60- to 1-min
	a	a	a	a	a
Logarithmic					
Gwangju	1.0250	1.0410	1.0540	1.0560	1.0800
Daegu	1.0300	1.0510	1.0710	1.0870	1.1120
Daejeon	1.0300	1.0350	1.0580	1.0740	1.0860
Busan	1.0190	1.0290	1.0330	1.0360	1.0400
Seogwipo	1.0150	1.0180	1.0200	1.0260	1.0090
Seoul	1.0350	1.0470	1.0680	1.0780	1.0950
Ulsan	0.9876	0.9382	0.8882	0.9138	0.9407
Incheon	1.0160	1.0260	1.0300	1.0340	1.0350
Chuncheon	1.0180	1.0380	1.0690	1.0880	1.1160
Average	1.0195	1.0248	1.0324	1.0436	1.0571

**Table 7:** Regression coefficients at different integration times for three empirical methods

Sites	5- to 1-min				10- to 1-min				20- to 1-min			
	a	b	c	d	a	b	c	d	a	b	c	d
Chebl and Rahman												
Gwangju	−17.310	0.73800	1.132	43.170	0.7784	1.0140	1.185	−8.234	−5.422	0.368	1.435	74.160
Daegu	1.365	−0.01046	−0.359	91.610	3.2690	−0.0086	−2.288	40.390	−4482.000	1.078	1.474	923.900
Daejeon	0.002	−0.00774	1.120	−5.060	−41.3400	0.9106	1.186	−5.278	1.313	1.064	1.271	−8.465
Busan	4.602	4.89800	1.118	−110.900	−15.6000	4.6630	1.139	−26.910	0.003	−0.431	0.987	8.064
Seogwipo	−1211.000	1.08700	1.085	433.600	0.1059	−0.0027	0.975	15.620	4.738	0.003	−3.521	0.013
Seoul	−2.586	0.05670	2.668	45.650	5.2160	2.7980	1.289	−245.800	−17.970	2.911	1.480	−453.400
Ulsan	0.173	0.76840	0.985	0.910	28.4200	0.0001	−27.470	−0.020	−0.141	3.589	0.702	571.600
Incheon	−386.700	0.93300	1.098	410.200	−3.3240	10.6400	1.157	−139.600	−44.460	2.476	1.153	−29.150
Chuncheon	−0.232	0.77940	1.075	1.790	−5719.0000	1.1340	1.254	966.700	−3289.000	1.033	1.491	895.900
Average	−179.076	1.02692	1.103	101.219	−637.9416	2.3498	−2.397	66.319	−870.327	1.343	0.719	220.291
Third order polynomial fit												
Gwangju	−0.0002625	0.0573600	−2.785	82.220	−0.00052400	0.11020000	−6.020	145.600	−0.00056630	0.11110000	−5.441	122.700
Daegu	0.0002814	−0.0614300	5.417	−92.990	0.00046860	−0.10280000	8.426	−156.500	0.00074800	−0.14550000	10.780	−197.700
Daejeon	−0.0003028	0.0663900	−3.393	94.290	−0.00043420	0.09233000	−4.862	120.900	−0.00075870	0.15210000	−8.134	177.700
Busan	0.0000145	−0.0011420	1.155	−6.092	−0.00019510	0.05392000	−3.261	106.100	−0.00017430	0.05149000	−3.158	104.900
Seogwipo	−0.0000840	0.0185500	−0.143	22.060	−0.00012210	0.02355000	−0.233	20.880	−0.00005324	0.00657900	1.028	−5.658
Seoul	−0.0000755	0.0212400	−0.352	26.140	0.00005647	−0.00577400	1.554	−15.610	0.00091930	−0.14930000	9.788	−173.800
Ulsan	0.0000548	−0.0239100	3.389	−62.770	0.00001800	−0.01718000	3.034	−56.490	0.00001578	−0.01106000	2.237	−30.670
Incheon	0.0000155	−0.0018370	1.180	−5.497	0.00131900	−0.27220000	19.770	−427.600	0.00226000	−0.44290000	29.850	−619.800
Chuncheon	0.0001048	−0.0207000	2.400	−27.110	−0.00040220	0.07533000	−2.927	63.840	0.00028430	−0.05043000	4.607	−70.850
Average	−0.0000282	0.0060579	0.763	3.361	0.00002050	−0.00473600	1.720	−22.098	0.00029720	−0.05310233	4.617	−77.020
Model 1												
Gwangju	−0.00000312800	0.00069080000	−0.04747	2.11800	−0.0000075820	0.0015920000	−0.104	3.283	−0.00000834800	0.00159900000	−0.094	2.886
Daegu	0.00000689500	−0.00153400000	0.10790	−1.23600	0.0000139400	−0.0030070000	0.205	−3.157	0.00002826000	−0.00545800000	0.339	−5.501
Daejeon	−0.00000463700	0.00104500000	−0.07348	2.70700	−0.0000065480	0.0014380000	−0.097	3.183	−0.00001869000	0.00363200000	−0.223	5.485
Busan	−0.00000001072	0.00000876500	0.00100	0.94870	−0.0000027210	0.0006997000	−0.054	2.330	−0.00000299900	0.00078050000	−0.060	2.494
Seogwipo	−0.00000094210	0.00019790000	−0.01171	1.22800	−0.0000006942	0.0000989500	−0.002	0.985	0.00000007355	−0.00008346000	0.011	0.761
Seoul	−0.00000084370	0.00018960000	−0.00897	1.12400	0.0000010330	−0.0002241000	0.021	0.456	0.00001399000	−0.00254100000	0.162	−2.378
Ulsan	0.00000095570	−0.00036440000	0.03484	0.11350	0.0000001916	−0.0001705000	0.020	0.473	0.00000006879	−0.00004612000	0.006	0.915
Incheon	0.00000034290	−0.00008152000	0.00787	0.78030	0.0000159800	−0.0033830000	0.236	−4.455	0.00002703000	−0.00539600000	0.352	−6.574
Chuncheon	0. 00000156600	−0.00033680000	0.02354	0.54900	−0.0000064710	0.0012230000	−0.067	2.204	0.00000916100	−0.00177400000	0.116	−1.118
Average	0.00000002201	−0.00002051722	0.00373	0.92583	0.0000007920	−0.0001925500	0.018	0.589	0.00000539404	−0.00103189778	0.068	−0.337

Sites	30- to 1-min				60- to 1-min			
	a	b	c	d	a	b	c	d
Chebl and Rahman								
Gwangju	13.050	−0.004	−12.270	0.101	0.001	−0.516	1.220	−25.240
Daegu	−5135.000	1.064	1.621	996.200	−90.760	0.533	2.165	454.900
Daejeon	0.005	−0.433	1.039	48.890	0.028	1.046	1.427	−26.920
Busan	−148.900	5.111	1.245	−245.200	−7.097	7.725	1.268	−252.400
Seogwipo	1.281	0.011	−0.039	93.690	−7.269	1.194	1.049	91.700
Seoul	0.014	−0.380	0.862	74.740	5.116	2.823	1.690	−605.900
Ulsan	58.940	3.524	0.717	556.200	5.590	0.226	0.132	−913.400
Incheon	−12.030	2.825	1.212	−182.800	0.710	1.017	1.171	−29.160
Chuncheon	−133.300	0.621	1.811	468.400	−3290.000	0.972	1.921	975.200
Average	−595.105	1.371	−0.423	201.136	−375.965	1.669	1.338	−36.802
Third order polynomial fit								
Gwangju	−0.00043350	0.08480000	−3.826	92.290	0.00028730	−0.03606000	3.077	−34.280
Daegu	−0.00029600	0.01206000	3.624	−94.220	−0.01276000	1.77700000	−76.950	1103.000
Daejeon	0.00266400	−0.37620000	18.600	−262.900	0.01033000	−1.50900000	73.190	−1119.000
Busan	−0.00002500	0.01923000	−0.783	46.990	0.00149900	−0.31010000	22.500	−490.800
Seogwipo	−0.00001095	−0.00142800	1.489	−12.950	−0.00003418	0.00503300	0.958	0.313
Seoul	0.00448700	−0.73490000	41.400	−733.200	−0.00012310	0.07349000	−4.201	103.3 00
Ulsan	0.00001323	−0.00829000	1.634	−7.223	0.00005752	−0.02056000	2.519	−27.090
Incheon	0.00407000	−0.80480000	53.800	−1144.000	0.00339900	−0.67410000	45.380	−963.900
Chuncheon	−0.00072290	0.10450000	−2.639	37.110	−0.00220900	0.29200000	−9.363	112.500
Average	0.00108288	−0.18944756	12.589	−230.900	0.00004962	−0.04469967	6.346	−146.217
Model 1								
Gwangju	−0.00000667000	0.00128300000	−0.0756	2.5780	0.00000877500	−0.00141400000	0.08129	−0.32120
Daegu	0.00001930000	−0.00427400000	0.2982	−5.0810	−0.00028400000	0.03620000000	−1.62800	23.86000
Daejeon	0.00001809000	−0.00205100000	0.0678	0.6327	0.00016300000	−0.02523000000	1.18100	−17.22000
Busan	−0.00000154700	0.00045000000	−0.0343	1.8360	0.00001705000	−0.00365300000	0.25900	−5.03200
Seogwipo	0.00000040390	−0.00013980000	0.0135	0.7558	−0.00000008082	−0.00000528700	0.00200	1.05700
Seoul	0.00006708000	−0.01158000000	0.6467	−11.0000	−0.00000258100	0.00088110000	−0.04907	1.81200
Ulsan	0.00000001307	0.00000295300	−0.0056	1.3830	0.00000025710	−0.00006486000	−0.00071	1.25200
Incheon	0.00005004000	−0.01016000000	0.6629	−13.4000	0.00004937000	−0.01013000000	0.66720	−13.62000
Chuncheon	−0.00000859100	0.00094650000	−0.0124	0.8382	−0.00003320000	0.00392800000	−0.11420	1.94300
Average	0.00001534655	−0.00283581633	0.1735	−2.3841	−0.00000904552	0.00005688367	0.04428	−0.69658

**Table 8:** Regression coefficients at different integration times for two empirical methods

Sites	5- to 1-min			10- to 1-min			20- to 1-min			30- to 1-min			60- to 1-min		
	a	b	c	a	b	c	a	b	c	a	b	c	a	b	c
Second order polynomial fit															
Gwangju	−0.004446	1.823	−25.820	−0.003514	1.858	−27.860	−0.00371	2.0450	−33.84	−0.003104	1.896	−26.65	0.01171	0.50630	10.52
Daegu	−0.002042	1.471	−11.390	−0.009772	2.609	−42.530	−0.01473	3.4670	−66.93	−0.039300	6.433	−143.10	−0.05993	8.99600	−206.50
Daejeon	−0.003097	1.594	−16.650	−0.008587	2.390	−39.260	0.00776	0.6405	8.51	0.065670	−5.184	150.50	0.08980	−7.30400	197.10
Busan	0.002808	0.809	3.585	0.003440	0.947	−6.733	0.00802	0.3478	13.52	0.013190	−0.309	34.91	0.03248	−3.06700	131.80
Seogwipo	−0.001491	1.379	−14.580	−0.006384	2.091	−35.820	−0.00658	2.0510	−30.51	−0.003942	1.671	−17.08	−0.00673	2.10900	−32.39
Seoul	0.002830	1.080	−9.231	0.006991	0.629	5.800	0.02882	−1.4320	55.99	0.066520	−5.381	159.20	0.05212	−2.98700	80.81
Ulsan	−0.003611	1.379	−5.937	−0.000816	0.740	21.750	−0.00003	0.1376	51.20	0.000134	0.135	50.15	0.00152	0.00574	49.92
Incheon	0.002014	0.868	2.733	0.025030	−2.231	106.900	0.04558	−4.9000	192.90	0.055120	−6.066	228.60	0.03645	−3.53000	144.60
Chuncheon	0.001093	1.008	−0.290	−0.007152	2.275	−35.180	−0.00322	2.1280	−29.99	−0.006939	2.839	−48.06	−0.04960	7.20700	−139.80
Average	−0.000660	1.268	−8.620	−0.000085	1.256	−5.881	0.00688	0.4983	17.87	0.016372	−0.441	43.16	0.01198	0.21512	26.23
Model 3															
Gwangju	−0.0100600	0.8060000	−25.820	−0.008008	0.8156	−27.86	−0.008836	0.8589	−33.84	−0.007133	0.825	−26.65	0.01649	0.3593	10.52
Daegu	−0.0041950	0.7158000	−11.390	−0.026380	0.9665	−42.53	−0.046380	1.1010	−66.93	−0.185200	1.365	−143.10	−0.37120	1.4520	−206.50
Daejeon	−0.0065930	0.7490000	−16.650	−0.022160	0.9260	−39.26	0.011720	0.4240	8.51	0.006760	−1.008	37.66	0.00552	−1.0050	55.55
Busan	0.0045870	0.4954000	3.585	0.005924	0.5496	−6.73	0.010380	0.2686	13.52	0.007718	−0.528	34.91	0.00377	−1.0040	62.33
Seogwipo	−0.0029810	0.6898000	−14.580	−0.015410	0.8661	−35.82	−0.015740	0.8570	−30.51	−0.008575	0.768	−17.08	−0.01632	0.8696	−32.39
Seoul	0.0051160	0.5974000	−9.231	0.010510	0.4184	5.80	0.006044	−1.0060	43.22	0.006662	−1.006	42.80	0.00712	−1.0070	45.80
Ulsan	−0.0072380	0.6880000	−5.937	−0.001300	0.4646	21.75	−0.000031	0.1218	51.20	0.000151	0.120	50.15	0.00152	0.0057	49.92
Incheon	0.0033710	0.5186000	2.733	0.003480	−1.0030	61.28	0.004942	−1.0050	36.59	0.004884	−1.004	39.78	0.00332	−1.0040	64.99
Chuncheon	0.0019310	0.5708000	−0.290	−0.017990	0.9045	−35.18	−0.007798	0.8777	−29.99	−0.019460	1.012	−48.06	−0.25730	1.3890	−139.80
Average	−0.0017847	0.6478667	−8.620	−0.007926	0.5454	−10.95	−0.005078	0.2776	−0.91	−0.021577	0.060	−3.29	−0.06745	0.0062	−9.95

The effectiveness of proposed model is observed from the coefficient of determination, R^2^, values as listed in Table 9. This statistical property of regression concerns the relationship between the PD of the parameter estimates and the true values of those parameters. The coefficient of determination, R^2^, describes the proportion of variance in measured data explained by the models. It is the portion of total variation in dependent variable that is explained by variation in independent variable (Steel and Torrie 1960). R^2^ ranges from 0 to 1, with higher values indicating less error variance whose values are summarized in Table 9. Out of ten mentioned empirical methods only Burgueno et al., second order polynomial fit, third order polynomial fit, Model 1, Model 2 and Model 3 values are listed because of dependability on statistical analyses for regression values.

**Table 9:** Coefficient of determination R^2^ as obtained from statistical program

Sites	5- to 1-min	10- to 1-min	20- to 1-min	30- to 1-min	60- to 1-min
	R^2^	R^2^	R^2^	R^2^	R^2^
Burgueno et al.					
Gwangju	0.9495	0.9519	0.9443	0.9538	0.8999
Daegu	0.9744	0.9400	0.9289	0.8449	0.8870
Daejeon	0.9519	0.8738	0.9361	0.8511	0.8334
Busan	0.9872	0.9863	0.9862	0.9888	0.9275
Seogwipo	0.9903	0.9730	0.9694	0.9901	0.7423
Seoul	0.9723	0.9793	0.9562	0.9128	0.9353
Ulsan	0.8929	0.7757	0.8040	0.8885	0.8956
Incheon	0.9892	0.9610	0.9245	0.9040	0.8475
Chuncheon	0.9742	0.9341	0.9643	0.9147	0.8118
Average	0.9647	0.9306	0.9349	0.9165	0.8645
Second order polynomial fit					
Gwangju	0.9556	0.9551	0.9542	0.9559	0.9006
Daegu	0.9757	0.9582	0.9574	0.9594	0.9519
Daejeon	0.9558	0.9079	0.9374	0.9014	0.8874
Busan	0.9877	0.9863	0.9877	0.9930	0.9517
Seogwipo	0.9913	0.9868	0.9851	0.9938	0.9799
Seoul	0.9722	0.9802	0.9610	0.9289	0.9392
Ulsan	0.9801	0.9097	0.7809	0.8828	0.9294
Incheon	0.9894	0.9801	0.9659	0.9481	0.8655
Chuncheon	0.9743	0.9508	0.9674	0.9217	0.9348
Average	0.9758	0.9572	0.9441	0.9428	0.9267
Third order polynomial fit					
Gwangju	0.9638	0.9677	0.9603	0.9603	0.9007
Daegu	0.9811	0.9653	0.9613	0.9597	0.9830
Daejeon	0.9694	0.9333	0.9459	0.9157	0.9605
Busan	0.9877	0.9877	0.9884	0.9930	0.9683
Seogwipo	0.9920	0.9898	0.9860	0.9938	0.9835
Seoul	0.9725	0.9803	0.9635	0.9446	0.9393
Ulsan	0.9922	0.9929	0.9896	0.9947	0.9797
Incheon	0.9894	0.9871	0.9749	0.9685	0.8754
Chuncheon	0.9756	0.9653	0.9682	0.9238	0.9491
Average	0.9804	0.9744	0.9709	0.9616	0.9488
Model 1					
Gwangju	0.9651	0.9703	0.9617	0.9617	0.9009
Daegu	0.9841	0.9707	0.9672	0.9611	0.9841
Daejeon	0.9746	0.9394	0.9540	0.9119	0.9587
Busan	0.9877	0.9880	0.9886	0.9931	0.9698
Seogwipo	0.9922	0.9900	0.9860	0.9938	0.9836
Seoul	0.9725	0.9804	0.9644	0.9482	0.9391
Ulsan	0.9957	0.9974	0.9936	0.9938	0.9806
Incheon	0.9894	0.9884	0.9762	0.9710	0.8783
Chuncheon	0.9754	0.9705	0.9681	0.9241	0.9504
Average	0.9819	0.9772	0.9733	0.9621	0.9495
Model 2					
Gwangju	0.9496	0.9503	0.9493	0.9524	0.9004
Daegu	0.9742	0.9386	0.9331	0.8393	0.8826
Daejeon	0.9521	0.8815	0.9369	0.8566	0.8381
Busan	0.9877	0.9861	0.9872	0.9906	0.9312
Seogwipo	0.9899	0.9751	0.9747	0.9912	0.9321
Seoul	0.9718	0.9801	0.9573	0.9122	0.9346
Ulsan	0.9778	0.8335	0.6145	0.3488	0.7466
Incheon	0.9893	0.9642	0.9285	0.9075	0.8498
Chuncheon	0.9743	0.9312	0.9623	0.9111	0.8012
Average	0.9741	0.9378	0.9160	0.8566	0.8685
Model 3					
Gwangju	0.9556	0.9551	0.9542	0.9559	0.9006
Daegu	0.9757	0.9582	0.9574	0.9594	0.9519
Daejeon	0.9558	0.9079	0.9374	0.8446	0.6838
Busan	0.9877	0.9863	0.9877	0.9930	0.7995
Seogwipo	0.9913	0.9868	0.9851	0.9938	0.9799
Seoul	0.9718	0.9801	0.9573	0.9122	0.7831
Ulsan	0.9801	0.9097	0.7809	0.8828	0.9294
Incheon	0.9894	0.8479	0.9349	0.8971	0.6771
Chuncheon	0.9743	0.9508	0.9674	0.9217	0.9348
Average	0.9757	0.9425	0.9403	0.9289	0.8489

As noted from Table 9, the average regression values obtained while applying Model 1 are 0.9819, 0.9772, 0.9733, 0.9621 and 0.9495 for 5-, 10-, 20-, 30- and 60- to 1-min conversion times respectively. These values are closer to unity as observed against the other applied models. Hence, the proposed model gives less chances of error variance.

### Evaluation of proposed method

In order to measure the goodness of fit of proposed model, paper present several error analyses. Mean, standard deviation (SD) and root mean square (RMS) values of error probability, ε(P), are gathered, where they are compared to the performance of the ITU-R P.837-6 (2012) model. Data for comparison of prediction methods are tabulated at fixed probability levels over decades where preferred values are 0.001, 0.002, 0.003, 0.005, 0.01, 0.02, 0.03, 0.05, and 0.1 % of time. Furthermore, mean, SD and RMS error values have been weighted over the probability levels of 0.001, 0.002, 0.003, 0.005, 0.01, 0.02, 0.03, 0.05, and 0.1 % of time, as recommended in ITU-R P.311-15 (2015).

Absolute percentage relative error figure is given as,

10 $$\upvarepsilon({\text{P}})_{\text{T}} = \frac{{{\text{R}}_{\text{e}} ({\text{P}})_{\text{T}} - {\text{R}}_{\text{m}} ({\text{P}})_{\text{T}} }}{{{\text{R}}_{\text{m}} ({\text{P}})_{\text{T}} }} \times 100\,(\% )$$

where R_e_(P)_T_ and R_m_(P)_T_ are the rain rate values of the estimated and the measured T-min integrated rainfall CDF, respectively, at the same probability level P, in the percentage interval 10^−3^ < P < 10^−1^ %.

RMS as defined by Owolawi and Afullo (2007),

11 $${\text{RMS}} = \left[ {(1/{\text{N}}) \times \sum\limits_{i = 1}^{\text{N}} {\left( {X_{est,i} - X_{mea,i} } \right)^{2} } } \right]^{1/2}$$

where N is the total number of available probability values, *X*_*est*_ and *X*_*mea*_ are the estimated and measured quantities respectively.

Similarly, SD, is calculated as,

12 $${\text{SD}} = \left[ {(1/{\text{N}}) \times \sum\limits_{i = 1}^{\text{N}} {\left( {{\varvec{\upvarepsilon}}({\mathbf{P}})_{\varvec{i}} -\varvec{\mu}} \right)^{2} } } \right]^{1/2}$$

where N is the total number of available probability values, ε(P)_*i*_ and *μ* are each error value and arithmetic mean of error quantities respectively.

The calculated error probabilities are presented in tabular form to accurately identify the obtained error values. The average error values thus obtained over all integration times using regional coefficient sets for each of the methods are listed in Table 10, which indicates that proposed model gives less relative error percentages which are <1 % for all conversion times. This is justified from lower values of SD and RMS calculation which is <7 % and 6 % in aggregate for all integration times respectively. In contrast, ITU-R P.837-6 (2012) produces higher error percentage of 5.19, 13.73, 43.23, 71.19 and 115.36 % for 5-, 10-, 20-, 30- and 60- to 1-min conversion time respectively. Hence, proposed model provides a better accuracy for all integration times. In addition, polynomial fits of third and second orders can be considered as a second and third preferred method because these models also show lower chances of error probabilities.

**Table 10:** Mean error obtained after testing over the interval [0.001–0.1 %]

Methods	5–1 min			10–1 min			20–1 min			30–1 min			60–1 min		
	Error $\upvarepsilon$ (%)	SD (%)	RMSE (%)	Error $\upvarepsilon$ (%)	SD (%)	RMSE (%)	Error $\upvarepsilon$ (%)	SD (%)	RMSE (%)	Error $\upvarepsilon$ (%)	SD (%)	RMSE (%)	Error $\upvarepsilon$ (%)	SD (%)	RMSE (%)
ITU-R P.837-6	5.19	11.35	13.57	13.73	12.26	19.02	43.28	19.18	42.25	71.19	25.28	67.55	115.36	36.92	106.06
Segal	1.18	7.83	8.56	4.09	17.80	20.44	3.85	12.93	12.28	2.21	10.52	9.73	1.31	10.52	10.00
Burgueno et al.	0.65	5.75	4.62	1.35	7.82	6.27	0.78	7.56	6.09	0.97	9.03	7.11	1.89	11.59	9.47
Chebil and Rahman	0.92	7.99	8.06	5.21	23.08	26.79	5.77	17.31	17.33	3.30	13.33	13.25	1.70	12.60	12.32
Logarithmic	0.66	8.41	8.24	2.06	18.93	20.56	1.47	16.57	14.73	1.52	16.32	14.71	1.57	17.77	16.12
Global coefficients	3.64	9.33	10.97	20.31	37.19	48.47	47.98	45.04	62.63	63.61	44.77	75.64	125.87	58.16	129.48
Second order polynomial fit	0.27	4.89	4.04	0.39	6.32	5.15	0.56	6.74	5.57	0.62	7.52	5.91	0.57	8.82	6.86
Third order polynomial fit	0.25	4.99	4.01	0.28	5.42	4.33	0.32	5.84	4.78	0.44	6.60	5.53	0.33	6.79	6.11
Model 1	0.19	4.43	3.52	0.27	4.94	3.88	0.27	5.30	4.24	0.42	6.02	4.88	0.17	6.24	5.60
Model 2	0.60	5.05	4.17	0.84	7.23	6.07	0.50	7.65	6.51	−0.08	10.23	8.28	1.51	11.49	9.24
Model 3	0.27	4.89	4.04	1.09	6.91	5.69	1.43	7.63	6.31	2.15	8.57	6.96	4.06	12.01	9.44

Furthermore, Table 11 presents the results of evaluation for average error probability using regional coefficient sets at 0.01 %, which is considered to be suitable time percentage for calculation of rain induced attenuation (ITU-R 2015) and is very crucial for system designers to obtain preliminary design of the satellite microwave link, satellite payload design and to have broad idea of rain attenuation for microwave engineers.

**Table 11:** Mean error obtained after testing over the 0.01 % of time

Methods	5–1 min		10–1 min		20–1 min		30–1 min		60–1 min	
	Error $\upvarepsilon$ (%)	RMSE (%)	Error $\upvarepsilon$ (%)	RMSE (%)	Error $\upvarepsilon$ (%)	RMSE (%)	Error $\upvarepsilon$ (%)	RMSE (%)	Error $\upvarepsilon$ (%)	RMSE (%)
ITU-R P.837-6	5.45	9.41	13.29	13.08	43.11	34.69	70.34	56.94	115.29	93.20
Segal	−2.74	5.48	−3.19	4.97	−5.54	5.27	−2.90	5.93	−2.66	5.39
Burgueno et al.	−2.36	3.75	−1.12	4.08	−3.01	4.00	0.69	5.87	0.23	5.56
Chebil and Rahman	−2.72	4.07	−1.85	4.76	−3.85	5.87	−2.10	7.44	0.21	5.56
Logarithmic	−3.36	4.37	−4.72	5.90	−7.05	6.28	−3.63	6.59	−3.12	5.99
Global coefficients	−0.78	4.35	6.62	6.24	19.75	15.90	40.20	32.46	102.34	82.44
Second order polynomial fit	−1.79	3.57	0.28	4.65	−2.75	4.22	0.15	6.18	−0.57	6.61
Third order polynomial fit	−1.06	4.68	0.10	5.10	−0.49	4.23	−0.37	5.82	−1.81	4.97
Model 1	−1.20	4.98	−0.66	5.16	−1.48	4.59	−0.31	4.97	−2.99	5.24
Model 2	−2.44	3.85	−1.81	4.57	−3.02	3.80	−1.00	6.84	0.55	5.23
Model 3	−1.78	3.56	0.94	4.40	−2.12	3.88	2.31	5.57	2.25	4.99

Considering the variability of the rain rate predictions at 0.01 % time exceedance, the third order polynomial fit does better result presenting the relative error values of −1.06, 0.1, −0.49, −0.37 and −1.81 % for 5-, 10-, 20-, 30- and 60–1-min conversion times respectively. This is supported by less value of RMS errors as presented in Table 11. Interesting, fair variability in error chances are observed from proposed model against the third order polynomial fit. Under this condition, proposed model can be consider as a second choice which is followed by second order polynomial fit as an third preferred model at 0.01 % of time. In contrary, ITU-R P.837-6 and global coefficients method provide high error values for all time percentages. Even though, these models shows low values of error probability at lower time percentage especially, 5- to 1-min conversion time, but still this error is higher than other prediction models. In order to verify the prediction performance of the models, relative error percentages along with SD and RMS values are calculated using the average coefficients sets. Table 12 shows the average values of relative error percentage, SD and RMS results as obtained by using the average coefficient sets for all the measurement sites.

**Table 12:** Mean error obtained after testing over the interval [0.001–0.1 %]

Methods	5–1 min			10–1 min			20–1 min			30–1 min			60–1 min		
	Error $\upvarepsilon$ (%)	SD (%)	RMSE (%)	Error $\upvarepsilon$ (%)	SD (%)	RMSE (%)	Error $\upvarepsilon$ (%)	SD (%)	RMSE (%)	Error $\upvarepsilon$ (%)	SD (%)	RMSE (%)	Error $\upvarepsilon$ (%)	SD (%)	RMSE (%)
ITU-R P.837-6	5.19	11.35	13.57	13.73	12.26	19.02	43.28	19.18	42.25	71.19	25.28	67.55	115.36	36.92	106.06
Segal	4.80	9.12	10.96	18.77	31.19	40.76	60.70	39.78	66.97	58.82	32.66	62.00	41.11	26.69	58.84
Burgueno et al.	72.98	15.54	65.80	284.49	109.89	312.35	557.54	211.91	569.30	599.16	200.34	599.28	658.11	186.55	618.01
Chebil and Rahman	1.75	8.93	10.27	−330.25	62.01	310.36	−29.65	18.88	45.46	−140.27	10.67	119.68	10.59	21.48	28.93
Logarithmic	1.01	9.08	10.47	5.42	28.75	36.63	9.15	28.53	36.07	7.06	23.66	31.11	6.08	21.48	29.96
Global coefficients	3.64	9.33	10.97	20.31	37.19	48.47	47.88	45.04	62.63	63.61	44.77	75.64	126.04	58.16	129.48
Second order polynomial fit	1.11	8.61	10.07	9.58	28.57	35.94	47.45	71.49	87.30	54.35	77.38	102.41	29.60	40.11	55.15
Third order polynomial fit	0.43	6.08	6.74	73.05	210.12	267.44	430.31	615.18	760.93	573.37	908.49	1210.06	−23.02	39.11	48.93
Model 1	0.89	7.81	9.06	1803.39	5106.40	6499.55	2850.68	4053.98	5046.73	2385.65	4108.23	5477.02	−590.12	829.88	1161.87
Model 2	0.71	9.39	11.06	13.44	49.90	63.71	32.72	59.61	75.06	13.79	43.35	60.33	4.28	31.95	49.08
Model 3	−3.38	8.01	10.50	−79.39	60.35	122.56	−108.42	29.39	109.67	−260.77	101.92	287.08	−506.55	180.25	530.17

As noted from Table 12, the proposed model, have good result for 5- to 1-min time conversion and error probability dramatically increases for other higher times conversion especially at 60- to 1-min conversion time where negative values of rain rate are obtained. This might be due to the exponential function that it includes. Similarly, ITU-R P.837-6, Burgueno et al., global coefficients and model 3 show higher error chances and are impracticable to use for 1-min rain rate derivation. In addition, Chebil and Rahman method and Third order polynomial fit produced increased error chances as per the increasing conversion times. Segal method and second order polynomial fit result in similar nature of error probabilities. Interestingly, logarithmic model is found to be best while considering the average coefficient sets because the error probabilities are lower as 1.01, 5.42, 9.15, 7.06 and 6.08 % for 5-, 10-, 20-, 30-, and 60- to 1-min conversion times respectively. This is verified from lower SD and RMS values of 9.08, 28.75, 28.53, 23.66 and 21.48 % along with 10.47, 36.63, 36.07, 31.11 and 29.96 % for 5-, 10-, 20-, 30- and 60- to 1-min respectively. Additionally, performances of models are graded at 0.01 % of time while using average coefficient sets whose results are shown in Table 13. As noted, third order polynomial fit produces less error chances as compared to other models.

**Table 13:** Mean error obtained after testing over the 0.01 % of time

Methods	5–1 min		10–1 min		20–1 min		30–1 min		60–1 min	
	Error $\upvarepsilon$ (%)	RMSE (%)	Error $\upvarepsilon$ (%)	RMSE (%)	Error $\upvarepsilon$ (%)	RMSE (%)	Error $\upvarepsilon$ (%)	RMSE (%)	Error $\upvarepsilon$ (%)	RMSE (%)
ITU-R P.837-6	5.45	9.41	13.29	13.08	43.11	34.69	70.34	56.94	115.29	93.20
Segal	0.47	4.39	7.27	6.49	36.59	28.83	43.00	33.92	31.52	38.90
Burgueno et al.	65.69	51.27	244.27	192.28	423.08	337.37	492.93	394.16	584.09	467.14
Chebil and Rahman	−2.00	4.66	−306.48	242.16	−40.98	32.63	−134.72	106.91	4.01	10.69
Logarithmic	−3.21	4.95	−5.03	5.73	−7.72	6.90	−3.95	6.42	−1.27	10.61
Global coefficients	−0.78	4.35	6.62	6.24	19.75	15.90	40.20	32.46	102.34	82.44
Second order polynomial fit	−2.32	4.60	−0.60	4.17	−0.92	6.93	4.58	9.04	9.90	14.51
Third order polynomial fit	−2.40	4.49	−0.65	4.55	−0.70	8.10	4.43	11.06	0.96	13.06
Model 1	−2.68	4.71	−2.52	4.38	−4.51	7.28	−5.52	8.09	−116.26	95.74
Model 2	−3.89	1.31	−5.03	5.37	−7.09	8.06	−11.60	9.56	−10.30	14.62
Model 3	−6.45	6.12	−59.29	47.49	−91.32	72.89	−200.87	161.22	−421.44	339.14

As indicated in Table 14, third order polynomial model produces relatively less error probabilities of −2.4, 0.65, 0.7, 4.43 and 0.96 % along with RMS values of 4.49, 4.55, 8.1, 11.06 and 13.06 % for 5-, 10-, 20-, 30- and 60- to 1-min respectively which indicates the model suitability. The second most suitable model shall be Logarithmic model with similar error probabilities values. In other hand, ITU-R P.837-6, Burgueno et al., Chebil and Rahman, global coefficients and model 3 result in greater error chances so these models are not preferable at this time percentage. In addition, Segal method gives better estimation from 5- to 1-min conversion time but the error chances increase as conversion times get increased. Proposed model 1 produces higher error chances at higher conversion time, especially 60- to 1-min and relative error values are <6 % for lower time conversion. Second order polynomial fit and model 2 result higher error values as compared to third order polynomial fit.

**Table 14:** Error obtained after testing over the interval [0.001–0.1 %] using regional coefficient sets

Methods	5–1 min			10–1 min			20–1 min			30–1 min			60–1 min		
	Error $\upvarepsilon$ (%)	SD (%)	RMSE (%)	Error $\upvarepsilon$ (%)	SD (%)	RMSE (%)	Error $\upvarepsilon$ (%)	SD (%)	RMSE (%)	Error $\upvarepsilon$ (%)	SD (%)	RMSE (%)	Error $\upvarepsilon$ (%)	SD (%)	RMSE (%)
ITU-R P.837-6	−6.59	9.63	12.64	−8.65	9.59	13.97	57.94	14.72	53.97	123.89	45.98	127.39	127.93	46.53	130.89
Segal	1.93	6.1	5.46	0.98	9.59	10.12	0.57	7.71	6.37	0.68	9.74	7.72	0.76	9.5	7.67
Burgueno et al.	0.59	6.44	5.85	2.14	9.97	9.47	0.47	8.5	6.74	0.26	13.42	10.29	0.5	13.14	10.88
Chebil and Rahman	0.98	6.44	5.84	0.94	9.47	9.94	1.12	10.28	8.75	0.62	8.67	7.14	2.4	14.59	13.32
Logarithmic	0.56	6.4	5.88	0.37	9.61	10.11	0.95	9.56	8.1	1.44	13.99	12.68	1.63	13.94	12.93
Global coefficients	0.23	6.42	5.95	7.8	11.02	13.63	16.8	9.91	14.07	27.26	15.96	22.01	75.85	21.75	60.89
Second order polynomial fit	0.26	6.61	5.6	0.58	9.29	8.09	0.61	8.1	6.67	1.12	11.21	8.37	1.15	12.46	8.95
Third Order polynomial fit	0.38	6.33	4.66	0.67	8.44	6.88	0.7	8.7	6.2	0.68	8.28	7.74	−0.4	4.74	5.32
Model 1	0.46	5.93	4.25	0.74	8.07	6.56	0.46	8.41	5.72	0.98	9.15	7.91	0.78	4.96	5.43
Model 2	0.99	6.5	5.84	2.7	9.96	9.18	0.42	8.27	6.69	0.28	13.03	10.1	0.54	12.86	10.73
Model 3	0.31	6.61	5.6	0.59	9.29	8.09	0.59	8.09	6.67	4.98	12.7	10.51	11.38	20.23	14.99

Furthermore, models performance is justified with the application of regional and average coefficient sets in Daejeon site. As previously mentioned, this site is also considered in ERA-40 data base. Daejeon is the fifth largest metropolis with elevation of about 77 m above sea level and average precipitation is above 300 mm during the month of July and August. The calculated rain rate from experimental 1-min rainfall amount in this site at 0.01 % of time is 79.8 mm/h. Performance of error analysis in this site is done by using regional coefficient sets and average coefficient sets which are listed in Tables 14 and 15 respectively while considering all integration times.

**Table 15:** Error obtained after testing over the interval [0.001–0.1 %] using average coefficient sets

Methods	5–1 min			10–1 min			20–1 min			30–1 min			60–1 min		
	Error $\upvarepsilon$ (%)	SD (%)	RMSE (%)	Error $\upvarepsilon$ (%)	SD (%)	RMSE (%)	Error $\upvarepsilon$ (%)	SD (%)	RMSE (%)	Error $\upvarepsilon$ (%)	SD (%)	RMSE (%)	Error $\upvarepsilon$ (%)	SD (%)	RMSE (%)
ITU-R P.837-6	−6.59	9.63	12.64	−8.65	9.59	13.97	57.94	14.72	53.97	123.89	45.98	127.39	127.93	46.53	130.89
Segal Method	1.63	6.5	6.15	8.75	10.51	13.36	34.74	11.31	27.2	33.97	15.08	26.07	25.94	13.24	20.59
Burgueno et al.	67.43	10.67	56.48	248.43	33.85	209.88	408.51	42.54	336.08	435.35	66.72	348.56	499.48	73.9	401.44
Chebil and Rahman	−1.31	6.24	5.98	−311.43	21	257.35	−40.49	7.73	37.81	−134.47	7.39	109.23	−4.29	13.45	15.17
Logarithmic	−2.15	6.22	6.22	−3.82	9.15	10.38	−9.06	8.92	13.18	−10.18	12.68	17.72	−9.38	12.71	17.46
Global coefficients	0.23	6.42	5.95	7.8	11.02	13.63	16.8	9.91	14.07	27.26	15.96	22.01	75.85	21.75	60.89
Second order polynomial fit	−2.16	6.53	6.03	0.13	9.78	10.29	−1.7	8.99	8.34	−2.55	13.48	13.48	−0.62	13.64	13.26
Third order polynomial fit	−1.89	6.32	5.87	−0.95	11.81	11.91	4.57	9.02	7.99	−10.76	14.5	14.3	−11.8	18.12	15.99
Model 1	−2.09	6.34	6.01	−1.48	12.09	12.85	−7.03	8.12	9.75	−15.66	11.55	19.17	−82.03	23.58	81.89
Model 2	−2.73	6.27	6.38	−3.57	10.08	11.15	−9.64	7.43	10.77	−20.54	9.71	21.6	−22.52	9.45	22.77
Model 3	−6.37	6.13	7.64	−60.36	5.44	53.45	−90.42	5.15	78.45	−184.06	14.8	156.31	−357.57	42.73	304.46

As noted from Table 14, proposed model 1 and third order polynomial fit exhibit relatively less error chances which is <1 % for all integration times. In contrast, ITU-R P.837-6 and global coefficients produce higher error probabilities which indicate that it is not suitable for 1-min conversion process.

As indicated by error data listed in Table 15, logarithmic model and second order polynomial fit produced low error values. Interestingly, second order polynomial fit gives less error percentages of −2.16, 0.13, −1.7, −2.55 and −0.62 % for 5-, 10-, 20-, 30- and 60- to 1-min conversion time. This is supported by less values of SD obtained as 6.53, 9.78, 8.99, 13.48 and 13.64 % along with RMS value of 6.03, 10.29, 8.34, 13.48 and 13.26 % for 5-, 10-, 20-, 30- and 60- to 1-min respectively. In contrary, Burgueno et al., Chebil and Rahman, ITU-R P.837-6 and model 3 resulted in higher relative error probabilities. This is justified through increased SD and RMS values as noted from Table 15.

Moreover, the regional and average coefficients are further tested at 0.01 % of time whose error values are depicted in Tables 16 and 17 respectively.

**Table 16:** Error obtained after testing at 0.01 % of time using regional coefficient sets

Models	5–1 min		10–1 min		20–1 min		30–1 min		60–1 min	
	Error $\upvarepsilon$ (%)	RMSE (%)	Error $\upvarepsilon$ (%)	RMSE (%)	Error $\upvarepsilon$ (%)	RMSE (%)	Error $\upvarepsilon$ (%)	RMSE (%)	Error $\upvarepsilon$ (%)	RMSE (%)
ITU-R P.837-6	−10.56	8.43	−12.68	10.12	54.44	43.44	117.52	93.78	121.25	96.76
Segal	−1.25	0.99	−3.20	2.55	0.20	0.16	6.41	5.11	8.49	6.78
Burgueno et al.	−3.00	2.39	−2.35	1.88	1.84	1.47	13.27	10.59	16.66	13.30
Chebil and Rahman	−2.55	2.03	−2.21	1.76	0.69	0.55	2.44	1.95	10.11	8.07
Logarithmic	−2.95	2.36	−3.69	2.94	0.85	0.68	7.75	6.18	10.29	8.21
Global coefficients	−3.22	2.57	3.56	2.84	17.62	14.06	38.71	30.89	99.88	79.70
Second order polynomial fit	−0.99	0.79	2.16	1.73	0.38	0.30	6.97	5.56	10.84	8.65
Third order polynomial fit	−3.13	2.50	−2.30	1.84	0.04	0.03	3.58	2.86	−1.27	1.01
Model 1	−4.62	3.69	−4.22	3.37	−1.76	1.40	4.54	3.62	−0.87	0.70
Model 2	−2.59	2.07	−1.18	0.94	1.06	0.84	12.80	10.21	16.34	13.04
Model 3	−0.94	0.75	2.17	1.73	0.36	0.29	11.75	9.37	13.77	10.98

**Table 17:** Error obtained after testing at 0.01 % of time using average coefficient sets

Models	5–1 min		10–1 min		20–1 min		30–1 min		60–1 min	
	Error $\upvarepsilon$ (%)	RMSE (%)	Error $\upvarepsilon$ (%)	RMSE (%)	Error $\upvarepsilon$ (%)	RMSE (%)	Error $\upvarepsilon$ (%)	RMSE (%)	Error $\upvarepsilon$ (%)	RMSE (%)
ITU-R P.837-6	−10.56	8.43	−12.68	10.12	54.44	43.44	117.52	93.78	121.25	96.76
Segal	−2.00	1.60	4.24	3.38	34.21	27.30	41.42	33.05	35.51	28.33
Burgueno et al.	61.61	49.17	234.47	187.10	413.62	330.07	486.52	388.24	576.39	459.96
Chebil and Rahman	−4.41	3.52	−300.65	239.92	−42.01	33.52	−134.33	107.20	3.14	2.51
Logarithmic	−5.59	4.46	−7.72	6.16	−9.33	7.44	−5.00	3.99	−2.10	1.68
Global coefficients	−3.22	2.57	3.56	2.84	17.62	14.06	38.71	30.89	99.88	79.70
Second order polynomial fit	−4.58	3.65	−3.36	2.68	−3.58	2.86	1.26	1.00	6.73	5.37
Third order polynomial fit	−4.74	3.78	−3.28	2.61	−2.47	1.97	2.09	1.66	8.83	7.04
Model 1	−5.01	4.00	−5.21	4.16	−6.27	5.00	−7.32	5.84	−90.30	72.06
Model 2	−6.30	5.03	−7.83	6.24	−9.10	7.26	−12.92	10.31	−11.99	9.57
Model 3	−8.59	6.86	−60.01	47.89	−91.15	72.74	−198.60	158.48	−411.29	328.21

As noted from Table 16, Third order polynomial fit and Segal method produced lower relative error values among which earlier method generate less error chances. This is indicated by Table 16 where Third order polynomial fit give values of −3.13, −2.3, 0.04, 3.58 and −1.27 % for 5-, 10-, 20-, 30- and 60- to 1-min conversion times respectively. This is supported by lower RMS values of 2.5, 1.84, 0.03, 2.86 and 1.01 % for 5-, 10-, 20-, 30- and 60- to 1-min conversion times respectively. In contrast, ITU-R P.837-6 and global coefficients generate larger error values for all integration times as indicted in Table 16.

As indicated in Table 17, logarithmic method and Second order polynomial fit give less error values among which former model shows less error variance for higher time integration. Although second and third order polynomial show better result at lower integration times especially 5-, 10-, 20- and 30- to 1-min but for higher integration time their error chances are increased. Hence, Logarithmic model is more preferable at 0.01 % of time. In contrary, ITU-R P.837-6, Burgueno et al., Chebil and Rahman methods and model 3 generate higher error values as depicted in Table 17 which signifies their unsuitability for prediction of 1-min rain rate at 0.01 % of time.

**Fig. 6:**
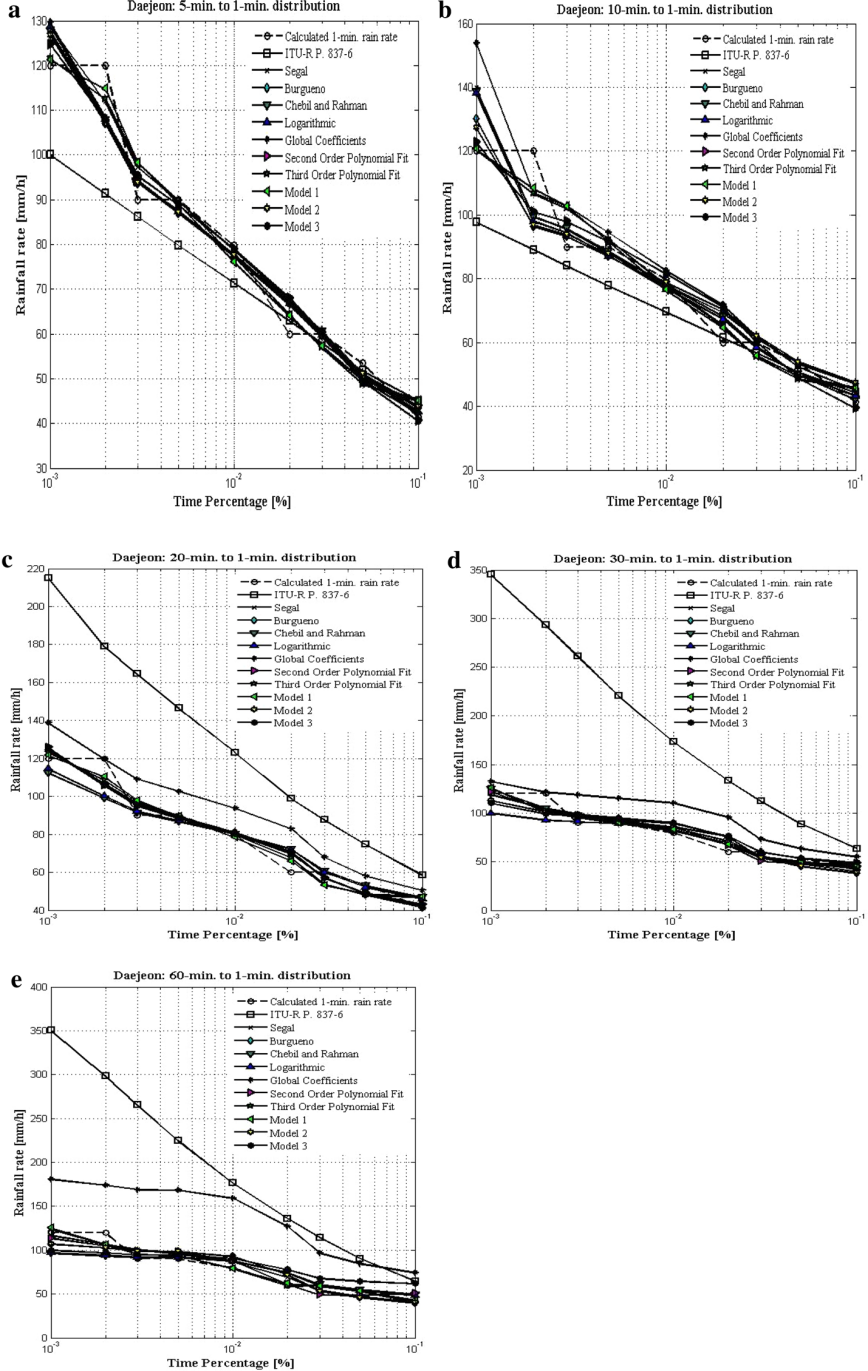
**a** 1-min rainfall rate compared with 5-min integration time rainfall rate data. **b** 1-min rainfall rate compared with 10-min integration time rainfall rate data. **c** 1-min rainfall rate compared with 20-min integration time rainfall rate data. **d** 1-min rainfall rate compared with 30-min integration time rainfall rate data. **e** 1-min rainfall rate compared with 60-min integration time rainfall rate data

Estimated 1-min rainfall rate curves for Daejeon Sites from 5-, 10-, 20-, 30- and 60- to 1-min conversion times are highlighted in Fig. 6a–e respectively. These plots indicate that empirical nature of models markedly follows the calculated 1-min rain rate pattern. Figure 6a, b indicate that ITU-R P.837-6 method under estimate calculated 1-min rain rate for lower times conversion especially 5- and 10- to 1-min. Additionally, Fig. 6c–e highlight the overestimation shown by ITU-R P.837-6 method and global coefficient approach against the calculated 1-min rain rate.

## Conclusion

The results obtained will serve as effective tools for communication system designer to understand the effect of rain in propagation medium. In this regard, this paper estimates the suitable empirical conversion model based on a decade long rainfall distribution data from KMA (2004–2013) over nine regions of the South Korea. Several rainfall rate conversion processes have been carried out for various integration times using these cumulative rainfall distribution. Rain rates exceeded for 0.001 < P < 0.1 % of the time was compared with prominent rain rate models analyzed in nine sites rainfall data. In addition, specific to 0.01 % time percentage value, several error metrics are evaluated. The performance criteria are based on the estimated statistics for Error percentage, SD and root mean square error over the nine different region’s rainfall data. The calculated 1-min rainfall rate from experimental 1-min rainfall amount is compared with ITU-R P.837-6 method and five existing empirical rainfall rate models along with five different polynomial fits. ITU-R P.837-6 method underestimate 1-min rain rate at lower integration times especially 5-, 10- to 1-min conversion time and overestimate at higher integration times mostly 20-, 30- and 60- to 1-min. Under regional coefficients set, proposed model 1, show better estimation of 1-min rain rate and at 0.01 % of time, third order polynomial fit hold satisfactory result with less error probabilities. Interestingly, proposed model 1 and third order polynomial fit shows same condition in Daejeon site. Similarly, while using average coefficients set, Logarithmic model hold better estimation of 1-min rain rate and at 0.01 % of time, Logarithmic model, second and third order polynomial fits give satisfactory result. Same condition is noted in Daejeon site too under average coefficients set approach. In overall, the paper emphasizes that ITU-R P.837-6 global 1-min rain rate estimation performance did not significantly reflect the South Korea’s local rainfall characteristics. On the basis of overall result, it can be concluded that the proposed model seems to provide a better and more reliable alternative to the ITU-R P.837-6 method for better estimation of 1-min rainfall rate. We hope this work will be milestone approach for system designer in improving communication satellite systems in the South Korea.

## References

[CR1] Abdulrahman AY (2010). A new rain attenuation conversion technique for tropical regions. Prog Electromagn Res B.

[CR2] Aris M (2013). 1-minute integrated rain rate statistics estimated from tropical rainfall measuring mission data. IEEE Antennas Wirel Propag Lett.

[CR3] Burgueno A, Puigcerver M, Vilar E (1988). Influence of rain gauge integration time on the rain rate statistics used in microwave communications. Antenna Telecommun.

[CR4] Capsoni C, Lorenzo L (2009). A physically based method for the conversion of rainfall statistics from long to short integration time. IEEE Trans Antennas Propag.

[CR5] Capsoni C (2009). A new prediction model of rain attenuation that separately accounts for stratiform and convective rain. IEEE Trans Antennas Propag.

[CR6] Chebil J, Rahman TA (1999). Rain rate statistical conversion for the prediction of rain attenuation in Malaysia. Electron Lett.

[CR7] Crane RK (1996). Electromagnetic wave propagation through rain.

[CR8] Emiliani LD, Luini L, Capsoni C (2008). Extension of ITU-R method for conversion of rain rate statistics from various integration times to 1-minute. Electron Lett.

[CR9] Emiliani LD, Luini L, Capsoni C (2009). Analysis and parameterization of methodologies for the conversion of rain-rate cumulative distributions from various integration times to one minute. IEEE Antennas Propag Mag.

[CR10] Emiliani L, Luini L, Capsoni C (2010) On the optimum estimation of 1-minute integrated rainfall statistics from data with longer integration time. In: Proceedings of the IEEE fourth European conference on antennas and propagation (EuCAP)

[CR11] Ippolito LJ (2012) Radiowave propagation in satellite communications. Springer Science & Business Media, New York

[CR12] Islam R, Abdulrahman YA, Rahman TA (2012). An improved ITU-R rain attenuation prediction model over terrestrial microwave links in tropical region. EURASIP J Wirel Commun Netw.

[CR13] ITU-R P.837-6 (2012) Characteristics of precipitation for propagation modeling. International Telecommunication Union, Geneva

[CR15] ITU-R P.311-15 (2015) Acquisition, presentation and analysis of data in studies of radiowave propagation. International Telecommunication Union, Geneva

[CR17] Jung M-W (2008). Empirical prediction models of 1-min rain rate distribution for various integration time. J Electromagn Eng Sci.

[CR18] Kestwal MC, Joshi S, Garia LS (2014) Prediction of rain attenuation and impact of rain in wave propagation at microwave frequency for tropical region (Uttarakhand, India). Int J Microw Sci Technol 2014:958498

[CR19] Khairolanuar MH (2014). New empirical conversion technique for 1-minute integration time of precipitation intensity in Malaysia. Aust J Basic Appl Sci.

[CR20] Korea Meteorological Administration (KMA). 61 16-gil Yeouidaebang-ro Dongjak-gu Seoul 07062, Republic of Korea http://web.kma.go.kr/eng/index.jsp

[CR21] Lee JH (1994). Conversion of rain rate distribution for various integration time. IEEE Trans Microw Theory Tech.

[CR22] Luini L, Capsoni C (2011). MultiEXCELL: a new rain field model for propagation applications. IEEE Trans Antennas Propag.

[CR23] Mandeep JS, Hassan SIS (2008). 60- to 1-min rainfall-rate conversion: comparison of existing prediction methods with data obtained in the Southeast Asia region. J Appl Meteorol Climatol.

[CR24] Matricciani E (2011) A mathematical theory of de-integrating long-time integrated rainfall and its application for predicting 1-min rain rate statistics. Int J Satell Commun Netw 29(6):501–530

[CR25] Ong JT, Zhu CN (1997) Effects of integration time on rain rate statistics for Singapore. In: Tenth international conference on antennas and propagation, vol 2 (Conf. Publ. No. 436). IET

[CR26] Owolawi PA (2011). Derivation of one-minute rain rate from five-minute equivalent for the calculation of rain attenuation in South Africa. PIERS Online.

[CR27] Owolawi PA, Afullo TJ (2007) Rainfall rate modeling and worst month statistics for millimetric line-of-sight radio links in South Africa. Radio Sci 42(6). doi:10.1029/2006RS003535

[CR28] Park Y-H, Lee J-H, Jambaljav N, Pack J-K (2002) Empirical study on the rain drop-size model for rain attenuation calculations. In: Proceedings of the URSI General Assembly. http://www.ursi.org/Proceedings/ProcGA05/pdf/F01P.4(01213).pdf

[CR16] Recommendations ITU (2015) Propagation data and prediction methods required for the design of terrestrial line-of-sight systems. ITU-R P.530-16

[CR29] Segal B (1986). The influence of rain gauge integration time on measured rainfall-intensity distribution functions. J Atmos Ocean Technol.

[CR14] Series P (2015) Propagation data and prediction methods required for the design of earth-space telecommunication systems. Recommendation ITU-R P.618-12

[CR30] Singh MSJ (2006). Rainfall rate conversion in Southeast Asia countries. Int J Infrared Millim Waves.

[CR31] Singh MandeepSingh, Jit KenjiTanaka, Iida Mitsuyoshi (2007). Conversion of 60-, 30-, 10-, and 5-minute rain rates to 1-minute rates in tropical rain rate measurement. ETRI J.

[CR32] Steel RGD, Torrie JH (1960). Principles and procedures of statistics.

[CR33] Uppala S (2005). The ERA-40 re-analysis. Q J R Meteorol Soc.

[CR34] www.mathworks.com, the mathworks, Inc. Protected by U.S. and international patents

